# The relationship between exposure to phthalate metabolites and adult-onset hypogonadism

**DOI:** 10.3389/fendo.2022.991497

**Published:** 2022-08-18

**Authors:** Zheng-Huan Liu, Lu-Chen Yang, Pan Song, Jun-Hao Chen, Zhu-Feng Peng, Qiang Dong

**Affiliations:** Department of Urology, Institute of Urology, West China Hospital, Sichuan University, Chengdu, China

**Keywords:** adult-onset hypogonadism (AOH), phthalate, di-(2-ethylhexyl) phthalate (DEHP), NHANES, exposure

## Abstract

**Objective:**

Adult-onset hypogonadism (AOH) is a common disease for males >40 years old and is closely associated with age-related comorbidities. Phthalates are compounds widely used in a number of products with endocrine-disrupting effects. However, little is known about the association between exposure to phthalates and the risk of AOH. Thus, we conducted this study to explore the potential association using the 2013-2016 National Health and Nutrition Examination Survey (NHANES) data.

**Method:**

Data on AOH and urinary phthalate metabolites were collected, and univariable and multivariable logistic regression analyses were adapted to evaluate the association. The concentrations of each metabolite were calculated and grouped according to their quartiles for the final analysis.

**Result:**

Finally, we found that the odds ratio (OR) increased with increased concentrations of di-(2-ethylhexyl) phthalate (DEHP) metabolites, including mono(2-ethyl-5-carboxypentyl) phthalate (MECPP), mono(2-ethyl-5-hydroxyhexyl) phthalate (MEHHP) and mono(2-ethyl-5-oxohexyl) phthalate (MEOHP). Simultaneously, a significant dose-dependent effect was also observed. The OR for the fourth quartile was highest among all three groups. Specifically, the ORs for the third quartile and fourth quartile were 1.774 and 1.858, respectively, in the MECPP group. For the MEHHP group, the OR increased from 1.580 for the second quartile to 1.814 for the fourth quartile. Similarly, the OR for the higher three quartiles varied from 1.424 to 1.715 in the MEOHP group.

**Conclusion:**

This study first revealed that there was a positive association between exposure to DEHP metabolites and the risk of AOH. These findings add limited evidence to study this topic, while further studies are needed to explain the potential molecular mechanisms.

## Introduction

The hypothalamic-pituitary-gonadal (HPG) axis plays an important role in many processes related to the development, maturation and aging of males (1). Any congenital or acquired disturbances of the HPG axis could lead to the clinical syndrome of hypogonadism. Male hypogonadism is defined as a disorder associated with decreased functional activity of the testes, with decreased production of sexual hormones (2). When hypogonadism is specifically associated with aging among adult males, adult-onset hypogonadism (AOH) is usually named late-onset hypogonadism (LOH). In heathy, young eugonadal males (defined as men younger than 30 years old with normal testosterone levels), serum testosterone levels vary between 10.4 and 36.4 nmol/L (300-1050 ng/dl), with a slightly gradual decline when older than 40 years old (3). Biochemical hypogonadism is defined as a serum total testosterone level < 11 nmol/L (317 ng/dl), and approximately 12, 20, 30 and 50% of men in their 50 s, 60 s, 70 s and 80 s, respectively, have biochemical hypogonadism (3). According to the biochemical hypogonadism criteria, AOH is a relatively common disease among males > 40 years of age, with a 2-15% prevalence within the general population (4). And AOH is usually closely associated with age-related comorbidities (5, 6). However, the mechanism of AOH still remains unclear.

Phthalates are a kind of synthetic chemicals applied to cosmetics, food packaging and medical products (7). Widespread application of phthalates could lead to pervasive human exposure (8, 9). Moreover, evidence based on experimental or observational data has shown the connection between exposure to phthalates and endocrine function, especially for sexual hormone dysregulation (10). Thus, we conducted this study to explore the relationship between exposure to phthalate metabolism and AOH using data from the National Health and Nutrition Examination Survey (NHANES) database in 2013-2016.

## Methods

### Study population

NHANES is a nationally representative survey among the noninstitutionalized American population, released in two-year cycles. NHANES was conducted by the CDC’s National Center for Health Statistics and was approved by the NCHS Research Ethics Review Board (ERB). We used the data from two cycles (2013-2014 and 2015-2016) of the NHANES database. The study sample was restricted to males older than 40 years old, without taking sexual hormone medications in the NHANES questionnaire, and had measured levels of urinary phthalate metabolite concentrations as well as serum total testosterone at the same time. We identified males with AOH using the definition of biochemical hypogonadism, which was a serum total testosterone level < 11 nmol/L (317 ng/dl). The data used in this study are publicly accessible on the NHANES website.

### Phthalate exposure

NHANES laboratories evaluated phthalate metabolite concentrations by using spot urine samples. The methods for exposure assessment have been described in detail in another study (11). Phthalate metabolites were grouped by parent phthalate molecule and the weight of the parent molecule according to NHANES guidelines. We divided phthalate metabolites into low molecular weight (LMW), high molecular weight (HMW), di-(2-ethylhexyl) phthalate (DEHP), diisononyl phthalate (DINP) and 2-cyclohexane dicarboxylic (DINCH) groups. In our study, only the phthalate metabolites detected in at least 75% of the samples were considered in the final analysis to avoid bias due to results below the limit of detection (LOD). The phthalate metabolites were grouped according to their quartiles for the final analysis.

### Statistical analysis

Data were analyzed using IBM SPSS Statistics 24 software (IBM Corp., Armonk, NY, USA), and a p value <0.05 was considered statistically significant. Data from the two cycles were combined for analysis *via* appropriate weighting methods (12). The environmental subsample B sample weights were used among all results. Univariate and multivariate logistic regression analyses were conducted to evaluate the association between AOH and exposure to phthalate metabolite concentrations. The potential confounding factors were identified based on previous studies, including age, race/ethnicity body mass index (BMI) and poverty-to-income ratio (PIR). PIR was grouped according to its quartiles. BMI was grouped into four categories based on the published standards of the National Institutes of Health (13).

## Result

Finally, we enrolled 1027 eligible cases in this study. **Table 1** demonstrates the characteristics of the study population in detail. The median age was 60 years old. Most of the population was non-Hispanic white (n=416). In our study, the median BMI was 28.10, indicating that most of the population was overweight. There were 354 cases with AOH, while 673 cases were without AOH. As shown in **Table 2**, there were 13 kinds of phthalate metabolites measured in this study. Three metabolites, including mono-isonoynyl phthalate (MNP), mono(2-ethylhexyl) phthalate and cyclohexane-1,2-dicarboxylic acid mono(hydroxy-isononyl) ester (MHINCH), were detected in less than 75% of the samples. Thus, we excluded these phthalate metabolites from the following analysis. **Table 3** summarizes the phthalate metabolite concentrations between different age groups. Metabolite concentrations did not vary significantly by age across most phthalate groups except for the monoethyl phthalate (MEP) and mono-n-butyl phthalate (MBP) groups. Men aged 40-60 had lower MEP concentrations than those aged >60 (31.55 vs 36.55, p=0.044). Similarly, men aged 40-60 had lower MBP (10.20 vs 11.20, p=0.009). The median exposure with corresponding interquartile ranges stratified by AOH is shown in **Table 4**. All DEHP-related metabolites were higher among men with AOH than those without, including mono(2-ethyl-5-carboxypentyl) phthalate (MECPP) (11.90 vs 9.80, p=0.001), mono(2-ethyl-5-hydroxyhexyl) phthalate (MEHHP) (8.00 vs 6.70, p=0.001) and mono(2-ethyl-5-oxohexyl) phthalate (MEOHP) (4.90 vs 4.20, p=0.003). In addition, men with AOH also had greater monocarboxynonyl phthalate (MCNP) levels than those without (12.50 vs 9.60, p=0.04). The other phthalate metabolites were not different between these two groups.

**Table 1:** Characteristics of study population, NHANES 2013-2016.

Characteristic	N	%	Median	(Interquartile range)
Total	1027	100		
Age (years)			60	(50, 69)
40-60	419	40.80		
≥60	536	59.20		
Race/ethnicity				
Mexican American	143	13.92		
Other Hispanic	110	10.71		
Non-Hispanic white	416	40.51		
Non-Hispanic black	222	21.62		
Non-Hispanic Asian	105	10.22		
Other/multi	31	3.02		
Poverty: income			2.25	(1.17, 4.47)
1^st^	232	22.59		
2^nd^	232	22.59		
3^rd^	233	22.69		
4^th^	232	22.59		
Missing	98	9.54		
BMI			28.10	(25.30, 31.80)
Normal (<25)	231	22.49		
Overweight (25-30)	418	40.70		
Obese (≥30)	362	35.25		
Missing	16	1.56		
Hypogonadism				
Yes	354	34.47		
No	673	65.53		

**Table 2:** Phthalate metabolites measured in NHANES, 2013-2016.

Phthalate metabolite	Grouping	LLOD (ng/mL)
Mono-ethyl phthalate (MEP)	LMW	1.2
Mono-isobutyl phthalate (MIBP)	LMW	0.8
Mono-n-butyl phthalate (MBP)	LMW	0.4
Mono(2-ethylhexyl) phthalate (MEHP)^a^	DEHP/HMW	0.8
Mono(2-ethyl-5-carboxypentyl) phthalate (MECPP)	DEHP/HMW	0.4
Mono(2-ethyl-5-hydroxyhexyl) phthalate (MEHHP)	DEHP/HMW	0.4
Mono(2-ethyl-5-oxohexyl) phthalate (MEOHP)	DEHP/HMW	0.2
Mono-isononyl phthalate (MNP)^a^	DINP/HMW	0.9
Monocarboxyoctyl phthalate (MCOP)	DINP/HMW	0.3
Monobenzyl phthalate (MBZP)	HMW	0.3
Monocarboxynonyl phthalate (MCNP)	HMW	0.2
Mono (3-carboxypropyl) phthalate (MCPP)	HMW	0.4
Cyclohexane-1,2-dicarboxylic acid mono(hydroxy-isononyl) ester (MHINCH)^a^	DINCH/HMW	0.4

LLOD, Lower limit of detection; LMW, low molecular weight; HMW, high molecular weight; DEHP, di-2-ethylhexyl phthalate; DINP, diisononyl phthalate; DINCH, 2-cyclohexane dicarboxylic; ^a^, detected in less than 75% of the samples.

**Table 3:** Phthalate metabolite group medians and interquartile ranges (IQR) stratified by age, NHANES 2013-2016. *<0.05.

	All	40 - 60	>60	p-value			
Phthalate metabolite groups (ng/ml)	Median	IQR	Median	IQR	Median	IQR	
LMW							
Mono-ethyl phthalate (MEP)	33.15	(13.70,120.8)	31.55	(12.40,113.6)	36.55	(15.80, 131.2)	0.044*
Mono-isobutyl phthalate (MIBP)	8.50	(4.20, 15.38)	8.60	(3.90, 15.08)	8.30	(4.40, 15.70)	0.902
Mono-n-butyl phthalate (MBP)	10.70	(5.40, 20.48)	10.20	(4.90, 18.83)	11.20	(5.90, 21.48)	0.009*
HMW							
Monobenzyl phthalate (MBZP)	3.40	(1.63, 8.00)	3.60	(1.60, 7.90)	3.40	(1.70, 8.18)	0.773
Monocarboxynonyl phthalate (MCNP)	10.65	(4.50, 27.28)	11.10	(4.23, 32.68)	10.05	(4.70, 26.05)	0.508
Mono (3-carboxypropyl) phthalate (MCPP)	1.40	(0.60, 3.40)	1.40	(0.53, 3.40)	1.50	(0.70, 3.38)	0.319
DEHP metabolites							
Mono(2-ethyl-5-carboxypentyl) phthalate (MECPP)	10.50	(5.63, 18.80)	10.35	(5.20, 18.68)	10.70	(6.10, 19.25)	0.290
Mono(2-ethyl-5-hydroxyhexyl) phthalate (MEHHP)	7.20	(3.60, 13.38)	7.05	(3.40, 13.30)	7.40	(3.70, 13.63)	0.510
Mono(2-ethyl-5-oxohexyl) phthalate (MEOHP)	4.60	(2.20, 8.40)	4.30	(2.00, 7.80)	4.70	(2.40, 8.85)	0.036*
DINP metabolites							
Monocarboxyoctyl phthalate (MCOP)	2.05	(1.10, 3.70)	2.10	(1.10, 4.10)	2.00	(1.10, 3.50)	0.392

*p < 0.05.

**Table 4:** Phthalate metabolite group medians and interquartile ranges (IQR) stratified by adult-onset hypogonadism, NHANES 2013-2016.

Phthalate metabolite groups (ng/ml)	Yes	No	p-value		
	Median	IQR	Median	IQR	
LMW					
Mono-ethyl phthalate (MEP)	34.90	(12.50, 133.1)	32.60	(14.70, 119.1)	0.947
Mono-isobutyl phthalate (MIBP)	8.80	(4.90, 15.85)	8.30	(3.90, 15.00)	0.198
Mono-n-butyl phthalate (MBP)	10.70	(5.70, 20.30)	10.70	(5.20, 20.60)	0.666
HMW					
Monobenzyl phthalate (MBZP)	3.70	(1.65, 8.00)	3.40	(1.60, 8.10)	0.811
Monocarboxynonyl phthalate (MCNP)	12.50	(4.85, 29.30)	9.60	(4.30, 26.20)	0.04*
Mono (3-carboxypropyl) phthalate (MCPP)	1.60	(0.70, 3.45)	1.40	(0.60, 3.30)	0.106
DEHP metabolites					
Mono(2-ethyl-5-carboxypentyl) phthalate (MECPP)	11.90	(7.25, 20.90)	9.80	(5.00, 17.70)	0.001*
Mono(2-ethyl-5-hydroxyhexyl) phthalate (MEHHP)	8.00	(4.40, 15.15)	6.70	(3.30, 12.50)	0.001*
Mono(2-ethyl-5-oxohexyl) phthalate (MEOHP)	4.90	(2.70, 9.00)	4.20	(2.00, 7.80)	0.003*
DINP metabolites					
Monocarboxyoctyl phthalate (MCOP)	2.10	(1.20, 4.00)	2.00	(1.00, 3.70)	0.134

LMW, low molecular weight; HMW, high molecular weight; DEHP, di-2-ethylhexyl phthalate; DINP, diisononyl phthalate; *p < 0.05.

The results of the univariable and multivariable logistic regression are shown in **Tables 5**, **6**, respectively. During the process, we divided the phthalate metabolites into four groups according to quartiles. The univariable logistic regression revealed significant associations between exposure to MCNP as well as DEHP metabolites (including MECPP, MEHHP, MEOHP) and AOH, consistent with the results shown in **Table 4**. In addition, there was also a significant relationship between monocarboxyoctyl phthalate (MCOP) exposure and AOH. However, after adjusting for age, PIR, and BMI, the associations between exposure to MCNP as well as MCOP and AOH disappeared. However, the relationships between exposure to DEHP metabolites were still significant. Men with higher MECPP, MEHHP or MHEOP concentrations had a higher risk of AOH. The odds ratio (OR) for the fourth quartile was highest among all three groups. Specifically, the ORs for the third quartile and fourth quartile were 1.774 and 1.858, respectively, in the MECPP group. For the MEHHP group, the OR increased from 1.580 for the second quartile to 1.814 for the fourth quartile. Similarly, the OR for the higher three quartiles varied from 1.424 to 1.715 in the MEOHP group.

**Table 5:** Univariable regression analysis.

Phthalate metabolite groups (ng/ml)	OR (95%CI)	*p*-Value
Mono-ethyl phthalate (MEP)		
<13.70	Ref	
13.70-33.15	0.695 (0.479, 1.008)	0.055
33.15-120.8	0.859 (0.596, 1.239)	0.417
≥120.8	0.922 (0.641, 1.326)	0.661
Mono-isobutyl phthalate (MIBP)		
<4.20	Ref	
4.20-8.50	1.387 (0.954, 2.018)	0.087
8.50-15.38	1.297 (0.889, 1.891)	0.177
≥15.38	1.398 (0.960,2.037)	0.080
Mono-n-butyl phthalate (MBP)		
<5.40	Ref	
5.40-10.70	1.369 (0.945, 1.985)	0.097
10.70-20.48	1.250 (0.861, 1.815)	0.242
≥20.48	1.138 (0.781, 1.658)	0.500
Monobenzyl phthalate (MBZP)		
<1.63	Ref	
1.63-3.40	0.936 (0.643, 1.361)	0.728
3.40-8.00	1.088 (0.756, 1.566)	0.650
≥8.00	0.994 (0.688, 1.435)	0.973
Monocarboxynonyl phthalate (MCNP)		
<4.50	Ref	
4.50-10.65	0.934 (0.638, 1.368)	0.726
10.65-27.28	1.590 (1.101, 2.296)	0.013*
≥27.28	1.256 (0.865, 1.823)	0.230
Mono (3-carboxypropyl) phthalate (MCPP)		
<0.60	Ref	
0.60-1.40	1.006 (0.682, 1.485)	0.975
1.40-3.40	1.407 (0.965, 2.051)	0.076
≥3.40	1.206 (0.819, 1.777)	0.343
Mono (2-ethyl-5-carboxypentyl) phthalate (MECPP)		
<5.63	Ref	
5.63-10.50	1.475 (1.002, 2.173)	0.049*
10.50-18.80	1.660 (1.136,2.428)	0.009*
≥18.80	1.965 (1.347, 2.867)	<0.001**
Mono (2-ethyl-5-hydroxyhexyl) phthalate (MEHHP)		
<3.60	Ref	
3.60-7.20	1.597 (1.088, 2.344)	0.017*
7.20-13.38	1.700 (1.160, 2.490)	0.006*
≥13.38	1.811 (1.237,2.652)	0.002*
Mono (2-ethyl-5-oxohexyl) phthalate (MEOHP)		
<2.20	Ref	
2.20-4.60	1.493 (1.018, 2.189)	0.040*
4.60-8.40	1.566 (1.068, 2.297)	0.022*
≥8.40	1.770 (1.211, 2.588)	0.003*
Monocarboxyoctyl phthalate (MCOP)		
<1.10	Ref	
1.10-2.05	1.517 (1.044,2.205)	0.029*
2.05-3.70	1.191 (0.808, 1.756)	0.378
≥3.70	1.419 (0.976, 2.063)	0.067

*p < 0.05; **p < 0.001.

**Table 6:** Multivariable regression analysis adjusted with age, PIR and BMI.

Phthalate metabolite groups (ng/ml)	OR (95%CI)	*p*-Value
Monocarboxynonyl phthalate (MCNP)		
<4.50	Ref	
4.50-10.65	0.900 (0.597, 1.358)	0.617
10.65-27.28	1.452 (0.979, 2.152)	0.064
≥27.28	1.217 (0.818, 1.810)	0.332
Mono (2-ethyl-5-carboxypentyl) phthalate (MECPP)		
<5.63	Ref	
5.63-10.50	1.268 (0.836, 1.922)	0.264
10.50-18.80	1.774 (1.182, 2.664)	0.006*
≥18.80	1.858 (1.238, 2.788)	0.003*
Mono (2-ethyl-5-hydroxyhexyl) phthalate (MEHHP)		
<3.60	Ref	
3.60-7.20	1.580 (1.048, 2.383)	0.029*
7.20-13.38	1.782 (1.183, 2.685)	0.006*
≥13.38	1.814 (1.205, 2.732)	0.004*
Mono (2-ethyl-5-oxohexyl) phthalate (MEOHP)		
<2.20	Ref	
2.20-4.60	1.424 (0.946, 2.141)	0.090
4.60-8.40	1.582 (1.049, 2.384)	0.028*
≥8.40	1.715 (1.138, 2.584)	0.010*
Monocarboxyoctyl phthalate (MCOP)		
<1.10	Ref	
1.10-2.05	1.473 (0.985, 2.203)	0.059
2.05-3.70	1.068 (0.704, 1.620)	0.758
≥3.70	1.279 (0.855, 1.913)	0.232

*p < 0.05.

## Discussion

In this study, a nationally representative cross-sectional study was adapted to explore whether urinary phthalate metabolites were associated with AOH. The results indicated a clear relationship between urinary phthalate metabolites, especially DEHP metabolite exposure and AOH. Simultaneously, a significant dose response trend was observed; that is, the OR increased with increasing DEHP metabolite concentrations compared with the lowest quartile.

AOH is defined as a clinical and biochemical syndrome featuring testosterone deficiency with signs or symptoms caused by HPG dysfunction (14). Biochemical hypogonadism is defined as a serum total testosterone level<317 ng/dl, which we applied in our study to identify AOH cases (3). However, the prevalence of AOH is still unclear. Many epidemiological studies vary in whether symptoms are considered and how androgen deficiency is defined. According to the data based on EMAS, the prevalence of hypogonadism was 13.8%. Moreover, the specific mechanism of AOH is also unclear. Phthalate is positively associated with endocrine dysfunction (10). A previous study showed that phthalate and its metabolite are associated with lower testosterone levels (15). Because AOH is characterized by lower total serum testosterone levels, we conducted this study to explore the association between AOH and phthalate metabolite exposure.

Phthalates are typically synthetic compounds widely applied as plasticizers, solvents and additives in many consumer products (16). Additionally, DEHP is one of the most widely used plasticizers and is regarded as an important endocrine-disrupting compound (17). Due to the widespread use of DEHP, humans tend to be exposed to DEHP and its metabolites (9). The metabolites of DEHP can be absorbed by the body in different ways, including dermal exposure, ingestion and inhalation (18). Importantly, various studies have revealed a positive association between exposure to DEHP and injury to the male reproductive system, including structure and function, especially in the testis (19). According to the European Food Safety Authority (EFSA) guidelines, male reproductive system injury could be caused by a daily DEHP exposure dose of 50 µg/kg, while there is more than a 300 µg/kg concentration of DEHP found in daily food (20). Furthermore, a previous study demonstrated that infertility patients have higher concentrations of DEHP and its metabolites in blood, urine and semen than normal males (19). Importantly, animal studies have revealed that DEHP and its metabolite could damage the structure of the testis, such as blood testicular barrier destruction and atrophy of seminiferous tubules, and furthermore lead to dysfunction of Leydig cells, including testosterone synthesis and secretion (21–24). As the results showed, the metabolites of DEHP were positively associated with a significantly higher risk of AOH, presenting a relevant dose-dependent effect. There are several potential mechanisms explaining the phenomenon. The previous study has shown the phthalates could inhibit stem Leydig cells differentiating into Leydig lineage cells while promote adipocyte differentiation (25). Besides, our study also indicates DEHP could induce the methylation of Sod2, Gpx1 and igf1, leading to testicular injury and dysfunction of Leydig cell (26). furthermore, DEHP could also induce apoptosis and autophagy of Leydig cell both *in vivo* and *in vitro*, through activating oxidative stress and p53 signaling pathway (27, 28). And testosterone is the major hormone in men, produced by Leydig cells, playing a vital role in maintaining normal reproductive and sexual function (29). For example, the spermatogenesis is totally dependent on testosterone level (30). When lacking of testosterone stimulation, the spermatogenesis would stay at meiosis stage (31). Thus, exposure to DEHP metabolites might increase the risk of AOH by damaging the structure and function of the male reproductive system, especially the testis. Further studies are needed to explore the specific mechanisms.

The strengths of this study include a relatively large, nationally representative sample with AOH and individually quantified urinary phthalate metabolites. Moreover, this analysis is the first study exploring the association between exposure to phthalates, especially DEHP metabolites, and the risk of AOH. And this finding could suggest that we might improve the management of AOH *via* decreasing the exposure of DEHP metabolites. However, there are still some limitations in our study. First, there is a lack of data on the symptoms caused by HPG dysfunction, such as erectile dysfunction, loss of morning erections and reduced sexual desire. Thus, we identified males with AOH using the definition of biochemical hypogonadism. Second, there is an inherent weakness due to its cross-sectional nature. Thus, further studies are needed to confirm the findings in our study and explain the related molecular mechanisms.

In summary, our study revealed a clear association between DEHP metabolite exposure and the risk of AOH in a dose-dependent manner. This brings us some insights, reducing the use of DEHP by replacement with structural analogues.

## Data availability statement

The original contributions presented in the study are included in the article/Supplementary Material. Further inquiries can be directed to the corresponding author.

## Author contributions

Z-HL and L-CY conceived and designed the study, collected and analyzed the data and wrote the manuscript. PS and J-HC analyzed the data. Z-FP and QD reviewed and edited the manuscript. All authors read and approved the manuscript.

## Funding

The work is supported by the Project of Science and Technology Department of Chengdu (grant no. 2021-YF05-00717-SN) and the Project of Science and Technology Department of Sichuan Province (2021YFS0117).

## Conflict of interest

The authors declare that the research was conducted in the absence of any commercial or financial relationships that could be construed as a potential conflict of interest.

## Publisher’s note

All claims expressed in this article are solely those of the authors and do not necessarily represent those of their affiliated organizations, or those of the publisher, the editors and the reviewers. Any product that may be evaluated in this article, or claim that may be made by its manufacturer, is not guaranteed or endorsed by the publisher.
